# Supplementary material for: SARS-CoV2 variant-specific replicating RNA vaccines protect from disease and pathology and reduce viral shedding following challenge with heterologous SARS-CoV2 variants of concern
Source: bioRxiv. 2021 Dec 13:2021.12.10.472134. Preprint. [Version 1] doi: 10.1101/2021.12.10.472134 (PMC8687464; doi:10.1101/2021.12.10.472134)
Supplement: 1 — Supplemental Figure 1. Effect of spike modifications on binding and neutralizing antibody responses in mice. C57BL/6 mice (n=5/group) were immunized with LION-formulated repRNA encoding the wild-type (WT), the prefusion-stabilized (PreF), the furin cleavage site-deleted (Furmut), or a combination of the PreF and Furmut modifications of the full-length spike of A.1 lineage SARS-CoV2. Vaccinations were administered on days 0 and 28, and serum collected on days 14, 28, and 38 assayed for (A) anti-spike binding IgG by enzyme linked immunosorbent assay and serum collected on days 28 and 38 assayed for (B) neutralizing antibody responses by 80% plaque reduction neutralization assay against A.1 SARS-CoV2 (WA-1 isolate). [file NIHPP2021.12.10.472134V1-supplement-1.pdf]

bioRxiv preprint doi: https://doi.org/10.1101/2021.12.10.472134; this version posted December 13, 2021. The copyright holder for this preprint (which was not certified by peer review) is the author/funder. This article is a US Government work. It is not subject to copyright under 17 USC 105 and is also made available for use under a CC0 license.

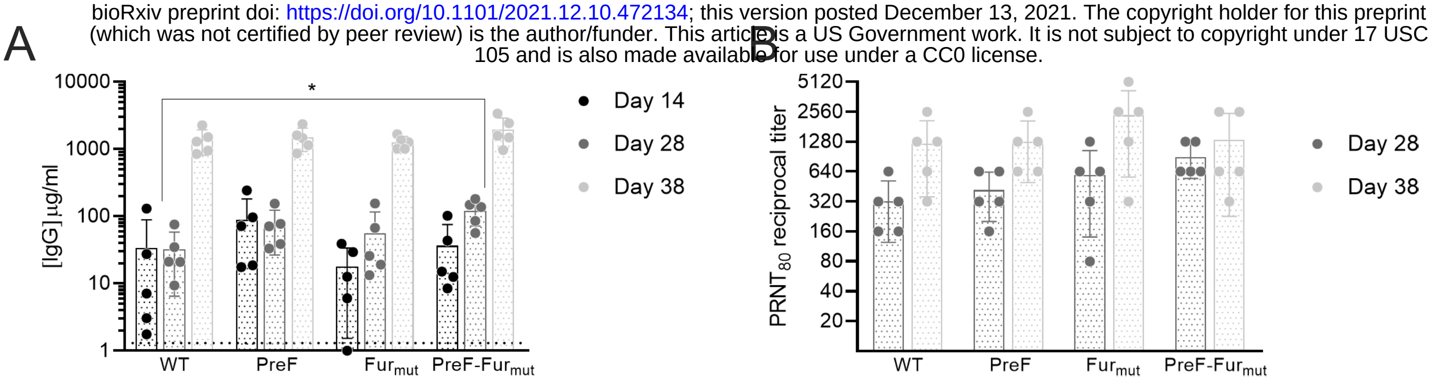

Supplemental Figure 1. Effect of spike modifications on binding and neutralizing antibody responses in mice. C57BL/6 mice (n=5/group) were immunized with LION-formulated repRNA encoding the wild-type (WT), the prefusion-stabilized (PreF), the furin cleavage site-deleted (Furmut), or a combination of the PreF and Furmut modifications of the full-length spike of A.1 lineage SARS-CoV2. Vaccinations were administered on days 0 and 28, and serum collected on days 14, 28, and 38 assayed for (A) anti-spike binding IgG by enzyme linked immunosorbent assay and serum collected on days 28 and 38 assayed for (B) neutralizing antibody responses by 80% plaque reduction neutralization assay against A.1 SARS-CoV2 (WA-1 isolate).
